# Tuberculosis Preventive Treatment Update — U.S. President’s Emergency Plan for AIDS Relief, 36 Countries, 2016–2023

**DOI:** 10.15585/mmwr.mm7311a1

**Published:** 2024-03-21

**Authors:** Aderonke S. Ajiboye, Stephanie O’Connor, Jonathan P. Smith, Sevim Ahmedov, William L. Coggin, Macarthur Charles, Smita Ghosh, Paul Pierre, Neha Shah, Richard A. Teran, Patrick K. Moonan, Anand Date

**Affiliations:** ^1^Division of Global HIV and Tuberculosis, Global Health Center, CDC; ^2^Tuberculosis Division, U.S. Agency for International Development, Washington, DC; ^3^Bureau of Global Health Security and Diplomacy, U.S. Department of State, Washington, DC; ^4^U.S. Military HIV Research Program, Walter Reed Army Institute of Research, Silver Spring, Maryland.

**Summary**

**What is already known about this topic?**

Tuberculosis (TB) is the leading cause of death among persons with HIV. TB preventive treatment (TPT), combined with antiretroviral treatment (ART), reduces TB-attributable deaths among persons with HIV. In 2018, the U.S. President’s Emergency Plan for AIDS Relief (PEPFAR) committed to offer TPT to eligible ART clients.

**What is added by this report?**

During October 2016–October 2023, approximately 13 million ART clients completed TPT in 36 countries. PEPFAR-supported programs achieved TPT completion rates up to 87%; initiation rates among clients who had been on ART <6 months (ART-naive) reached 59%.

**What are the implications for public health practice?**

Continued efforts are needed to maximize TPT coverage, especially for ART-naive clients. Short-course regimens, patient-centered care, and modernized medical record systems might help accomplish this goal.

## Abstract

Tuberculosis (TB) is the leading cause of death among persons with HIV. In 2022, an estimated 167,000 TB-related deaths occurred globally among persons with HIV. TB preventive treatment (TPT) helps prevent TB disease and is recommended for persons at high risk for developing TB, including those with HIV. TPT, when taken with antiretroviral treatment (ART), can reduce TB-attributable deaths among persons with HIV. In 2018, the U.S. President’s Emergency Plan for AIDS Relief (PEPFAR) program committed to offer one course of TPT to all eligible clients receiving ART. This analysis describes trends in TPT initiation and completion among PEPFAR-supported programs in 36 countries in Africa, Central and South America, and Asia during fiscal years (FYs) 2017–2023. Overall, TPT initiation rates peaked in FY19, a possible sign of programmatic saturation. TPT initiation among clients who had been on ART <6 months reached 59%, and overall completion rates up to 87% were reported. Approximately 13 million persons with HIV have completed TPT since FY17, but widespread adoption of shorter regimens, patient-centered approaches, and electronic medical record systems might be needed to ensure full TPT coverage. Through PEPFAR’s partnership with national HIV programs, TPT has become the standard of care for persons with HIV.

## Introduction

In 2022, an estimated 167,000 persons living with HIV experienced tuberculosis (TB)–related deaths globally, making TB the leading cause of death in this group (*1*). World Health Organization–recommended TB preventive treatment (TPT) regimens (*2*) reduce the risk for TB disease and TB-attributable deaths among persons with HIV.^†^ TPT is recommended for persons living with HIV once active TB disease has been ruled out, even when latent TB infection status is unknown (*2*). TPT has historically consisted of once-daily isoniazid for 6 or 9 months; shorter 1- and 3-month rifapentine-based regimens are now available (*2*). At the 2018 United Nations General Assembly High-Level Meeting (UNHLM) on TB, member countries agreed to provide TPT to 6 million persons with HIV by 2022.**^§^** In alignment with this announcement, the U.S. President’s Emergency Plan for AIDS Relief (PEPFAR) committed to offer at least one course of TPT to all eligible clients receiving antiretroviral treatment (ART), including pregnant women.^¶^ This report summarizes PEPFAR’s global progress on providing TPT to all ART clients in PEPFAR-supported programs, a cohort that includes approximately 19 million persons with HIV.

## Methods

Data were collected through PEPFAR monitoring, evaluation, and reporting indicators.** Data were collected at 6-month intervals and disaggregated by age group (<15 and ≥15 years), sex, and HIV treatment status (<6 months on ART [ART-naive] and ≥6 months on ART [ART-experienced]). Semiannualized TPT initiation and completion rates were calculated among persons on ART in 36 PEPFAR-supported programs that reported TPT data at any time during fiscal years (FYs) 17–23.^††^ Initiation rates were calculated through FY23 quarter (Q) 2, and completion rates were calculated through FY23 Q4. TPT initiation rates were calculated as the number of TPT initiations in a 6-month period divided by the number of ART clients on treatment at the end of that period. Analysis of TPT initiation rates among ART-naive clients included only those initiating TPT within 6 months of ART initiation. TPT completion rates were calculated as the number of TPT completions in a 6-month period divided by the number of TPT initiations in the previous reporting period. TPT initiation and completion rates were aggregated across all PEPFAR-supported programs. Mann-Whitney-U tests (α = 0.05) were used to assess stratum-specific differences in TPT initiation and completion rates. Data were analyzed using R software (version 4.3.2; R Foundation). This activity was reviewed by CDC, deemed not research, and conducted consistent with applicable federal law and CDC policy.^§§^

## Results

### Tuberculosis Preventive Treatment Initiation

The number of PEPFAR-supported countries that reported TPT data more than doubled during the analytic period (17 in FY17 and 36 in FY21). Overall, 16,832,651^¶¶^ TPT initiations were reported during FY17–23. The number of persons who initiated TPT increased by an average of 26% between each semiannual period during FY17–19 (Table). In the following semiannual period (FY20 Q2), the number of persons who initiated TPT decreased by 12%. TPT initiations began increasing again after FY20 Q2 and reached an all-time high in FY21 Q4 (1,802,814). Since then, the number of persons initiating TPT per year has declined. The overall increase in TPT initiations until FY20 was also reflected in initiation rates. During FY17–18, the semiannualized TPT initiation rates among all persons receiving ART (ART-naive and -experienced) ranged between 4% and 6% (Table). The TPT initiation rate peaked in FY19 Q4 (11%) and has since declined to 5% as of FY23 Q2. By contrast, the TPT initiation rate among ART-naive clients rose through FY22 Q4, from 17% in FY18 Q4 to 59% in FY22 Q4, before dropping to 53% in the most recent period assessed.

**TABLE:** Tuberculosis preventive treatment initiations* among persons with HIV — 36 U.S. President’s Emergency Plan for AIDS Relief–supported countries, October 2016–March 2023

Semiannual period^†^	Date range	Persons on ART	Persons on ART initiating TPT, no. (%)	Persons newly on ART	Persons newly on ART initiating TPT, no. (%)
FY17 Q2	Oct 2016–Mar 2017	11,726,101	654,161 (6)	—	—
FY17 Q4	Apr–Sep 2017	13,245,470	562,345 (4)	—	—
FY18 Q2	Oct 2017–Mar 2018	13,235,513	750,282 (6)	—	—
FY18 Q4	Apr–Sep 2018	14,769,349	750,431 (5)	1,437,294	243,744 (17)
FY19 Q2	Oct 2018–Mar 2019	13,433,062	1,192,952 (9)	1,236,576	308,431 (25)
FY19 Q4	Apr–Sep 2019	15,686,915	1,784,375 (11)	1,426,483	449,964 (32)
FY20 Q2	Oct 2019–Mar 2020	15,480,007	1,577,641 (10)	1,287,300	529,323 (41)
FY20 Q4	Apr–Sep 2020	17,383,890	1,651,619 (10)	1,194,562	579,085 (48)
FY21 Q2	Oct 2020–Mar 2021	17,248,709	1,750,779 (10)	1,173,027	615,747 (52)
FY21 Q4	Apr–Sep 2021	17,931,849	1,802,814 (10)	1,107,204	643,338 (58)
FY22 Q2	Oct 2021–Mar 2022	18,573,343	1,473,871 (8)	1,041,786	609,569 (59)
FY22 Q4	Apr–Sep 2022	19,238,096	1,311,024 (7)	1,007,261	591,548 (59)
FY23 Q2	Oct 2022–Mar 2023	19,472,835	1,014,421 (5)	934,074	494,023 (53)

**Abbreviations:** ART = antiretroviral treatment; FY = fiscal year; PEPFAR = U.S. President’s Emergency Plan for AIDS Relief; Q = quarter; TPT = tuberculosis preventive treatment.

* TPT initiation rates were calculated as the number of TPT initiations in a 6-month period divided by the number of ART clients on treatment at the end of that period. Analysis of TPT initiation rates among ART-naive (newly on ART) clients include only those initiating TPT within 6 months of ART initiation. TPT initiations are reported in the period after the 6-month period when they occur. TPT initiations reported in FY17 Q2 were included in total counts but initiation rates for that period were not calculated, because the denominator (number of persons on ART) was outside the temporal scope of this report.

^†^ The U.S. government FY runs October–September. In alignment with the PEPFAR reporting calendar, Q2 for semiannual metrics represents October–March of the following calendar year, and Q4 covers April to September.

### Tuberculosis Preventive Treatment Completion

**FIGURE 1:**
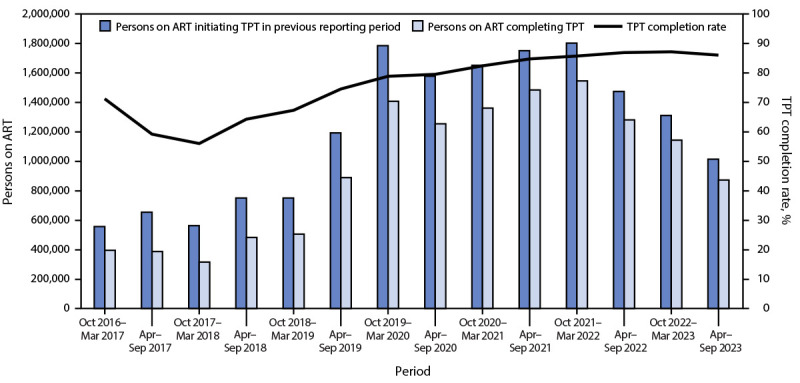
Tuberculosis preventive treatment completions* among persons on antiretroviral treatment — 36 U.S. President’s Emergency Plan for AIDS Relief–supported countries, October 2016–September 2023 **Abbreviations:** ART = antiretroviral treatment; TPT = tuberculosis preventive treatment. * TPT completion rates were calculated as the number of TPT completions in a 6-month period divided by the number of TPT initiations in the previous reporting period.

Overall, 13,323,186 persons with HIV have completed TPT in PEPFAR-supported programs that report TPT data. TPT completion rates steadily increased from 56% in FY18 Q2 to 87% in FY23 Q2, before dropping to 86% in FY23 Q4 (Figure 1).

### Differences by Sex, Age, and HIV Treatment Status

**FIGURE 2:**
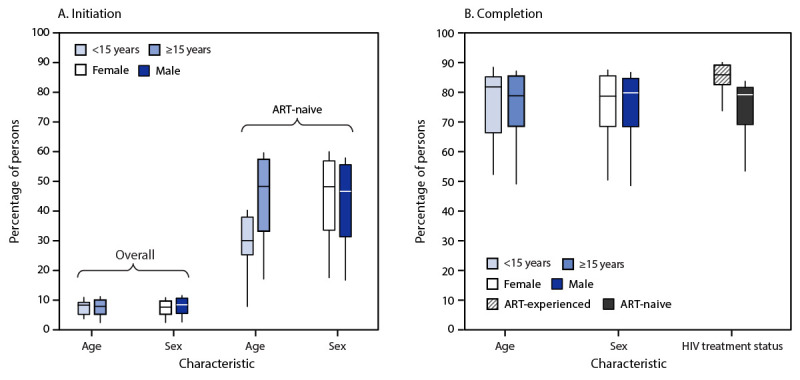
Differences* in tuberculosis preventive treatment initiation (A)^†^ and completion (B)^§^ rates among persons on antiretroviral treatment, by age, sex, and HIV treatment status^¶^ — 36 U.S. President’s Emergency Plan for AIDS Relief–supported countries, October 2016–September 2023** **Abbreviations:** ART = antiretroviral treatment; TPT = tuberculosis preventive treatment. * Mann-Whitney-U test (α = 0.05) assessed stratum-specific differences in TPT initiation and completion rates. ^†^ TPT initiation rates were calculated as the number of TPT initiations in a 6-month period divided by the number of ART clients on treatment at the end of that period. Analysis of TPT initiation rates among ART-naive clients include only those initiating TPT within 6 months of ART initiation. P-values for differences by characteristic were age (overall): p = 0.72; sex (overall): p = 0.48; age (ART-naive): p = 0.04; and sex (ART-naive): p = 0.53. ^§^ TPT completion rates were calculated as the number of TPT completions in a 6-month period divided by the number of TPT initiations in the previous reporting period. P-values for differences by characteristic were age: p = 0.98; sex: p = 0.98; and HIV treatment status: p<0.01. ^¶^ Persons who initiated TPT within 6 months of ART initiation were included in the analysis of TPT initiation rates among ART-naive clients; those on ART for ≥6 months when initiating TPT were ART-experienced. ** Whiskers display the full range of values for each metric. Boxes display IQRs, with median values indicated by a horizontal line within the box.

No statistically significant differences existed in overall TPT initiation or completion rates between sex and age groups (Figure 2). Among ART-naive clients, initiation rates were lower among those aged <15 years than among those aged ≥15 years (32% and 51%, respectively; p = 0.04). TPT completion rates were lower among ART-naive clients compared with ART-experienced clients (79% and 86%, respectively; p<0.01).

## Discussion

PEPFAR has supported the widespread integration of TPT as part of the HIV standard of care. As a result, approximately 13 million persons with HIV have completed TPT. These TPT completions meaningfully contributed to the 2018 UNHLM target for TPT among persons with HIV, the only UNHLM target achieved (*1*).

TPT initiation rates among ART-naive clients help monitor adoption of TPT into routine practice and are expected to be higher than initiation rates among ART-experienced clients, who might have already completed a course of TPT. Trends in overall initiations provide insight into TPT scale-up over time because climbing initiation rates would be expected when programs are rolling out TPT to the existing patient population. Declining overall TPT initiation rates over time might suggest programmatic saturation, in which all eligible ART clients have already received TPT. Importantly, PEPFAR program data cannot be used to directly measure saturation because these data are not person-level, and TPT completion was not collected before FY17.

Although overall TPT initiation rates trended downward, the percentage of ART-naive clients who received TPT increased. These trends might be indicative of a prioritization of TPT provision for those newly initiated on ART. At the country level, TPT coverage might vary by clinical guidance, eligibility, or supply chain mechanisms. Initiation rates were similar by age and sex, suggesting these factors did not play a major role in TPT initiation overall. However, lower initiation rates were noted among younger ART-naive clients compared with those aged ≥15 years.

Findings from this analysis were consistent with other reports that found lower TPT completion rates among ART-naive clients (*3*). Lower TPT completion rates have been found to be associated with perceived stigma (*4*), which might be higher among those recently diagnosed with HIV (*5*). High levels of stigma related specifically to TPT have also been documented (*6*), and other barriers to TPT completion such as pill burden (*7*), lack of health education, and distance to health facilities (*8*) can affect ART-naive clients differently.

### Limitations

The findings in this report are subject to at least four limitations. First, PEPFAR-wide results represent a diverse range of settings and populations, and the number of countries reporting TPT data varied over time.*** As a result, aggregated values might not reflect trends in individual countries or subnational units, and trends over time are not representative of a true cohort. Second, because TPT completion is often measured on the basis of pill dispensation and self-report rather than direct observation or biomarker monitoring, completion rates might be overestimated. Third, the data used for this analysis were collected in a programmatic setting for monitoring purposes. Data quality might fall short of the accuracy and precision of data collected for clinical studies or in other research settings. Finally, no person-level data were available, and data were reported in broad age bands (<15 and ≥15 years), precluding more specific analyses.

### Implications for Public Health Practice

The steady increase in TPT completion rates suggests substantial improvements in HIV and TB service delivery, monitoring, and reporting practices. However, opportunities remain to ensure full TPT coverage and maximize the impact of TPT in reducing TB morbidity and mortality. An ongoing need exists to ensure all ART-naive clients receive the requisite support to access and complete a full course of TPT. Patient-level electronic medical record systems could be developed and expanded to better identify underserved geographic areas and subpopulations and to monitor outcomes over time. Offering patient-centered approaches to treatment delivery can help make health care access a positive and convenient experience for clients by aligning service delivery with their preferences and needs (*9*). Increasing access to short-course regimens for all could improve completion rates (*2*), and ensuring availability of pediatric TPT formulations might increase coverage among persons with HIV aged <15 years. Promoting the use of digital adherence tools, such as mobile telephone applications and electronic sensor-enabled pill boxes (*10*), could help support clients throughout the course of treatment. Finally, further population-level analyses could help determine whether TPT implementation has been associated with reductions in TB incidence and TB-attributable deaths in settings where broad TPT coverage was achieved. Importantly, lessons learned from TPT implementation in PEPFAR-supported programs might prove useful for TPT provision among other populations at risk, including household contacts of persons with TB.

## Footnotes

* These authors contributed equally to this report.

†
https://www.cdc.gov/globalhivtb/who-we-are/success-stories/success-story-pages/scaling-tpt-ethiopia.html

§
https://www.who.int/publications/m/item/political-declaration-of-the-un-general-assembly-high-level-meeting-on-the-fight-against-tuberculosis

¶
https://na.usembassy.gov/wp-content/uploads/sites/132/PEPFAR-COP18-Guidance_FINAL-1.pdf

**
https://help.datim.org/hc/article_attachments/10003735798420

†† The U.S. government FY runs October–September. In alignment with the PEPFAR reporting calendar, Q2 for semiannual metrics represents October–March of the following calendar year, and Q4 covers April–September.

§§ 45 C.F.R. part 46.102(l)(2), 21 C.F.R. part 56; 42 U.S.C. Sect. 241(d); 5 U.S.C. Sect. 552a; 44 U.S.C. Sect. 3501 et seq.

¶¶ This value includes 555,936 TPT initiations that were reported in FY17 Q2 but occurred in FY16, in accordance with PEPFAR's standard indicator definition for TPT. To calculate TPT initiation rates, the number of TPT initiations must be aligned with the number of persons on ART in the previous reporting period. TPT initiations reported in FY17 Q2 were included in total counts, but initiation rates for that period were not calculated because the denominator (number of persons on ART) was outside the temporal scope of this report.

*** Because of ongoing data quality assessments, data from one country that has historically reported a large number of TPT completions were not included in the most recent period assessed (FY23 Q4).
